# Open-Source Electronic Health Record Systems for Low-Resource Settings: Systematic Review

**DOI:** 10.2196/medinform.8131

**Published:** 2017-11-13

**Authors:** Assel Syzdykova, André Malta, Maria Zolfo, Ermias Diro, José Luis Oliveira

**Affiliations:** ^1^ University of Aveiro Department of Electronics, Telecommunications and Informatics (DETI) / Institute of Electronics and Informatics Engineering of Aveiro (IEETA) Aveiro Portugal; ^2^ BMD Software Aveiro Portugal; ^3^ Institute of Tropical Medicine Antwerp Belgium; ^4^ University of Gondar Gondar Ethiopia

**Keywords:** electronic health record, EHR, software, eHealth, open source

## Abstract

**Background:**

Despite the great impact of information and communication technologies on clinical practice and on the quality of health services, this trend has been almost exclusive to developed countries, whereas countries with poor resources suffer from many economic and social issues that have hindered the real benefits of electronic health (eHealth) tools. As a component of eHealth systems, electronic health records (EHRs) play a fundamental role in patient management and effective medical care services. Thus, the adoption of EHRs in regions with a lack of infrastructure, untrained staff, and ill-equipped health care providers is an important task. However, the main barrier to adopting EHR software in low- and middle-income countries is the cost of its purchase and maintenance, which highlights the open-source approach as a good solution for these underserved areas.

**Objective:**

The aim of this study was to conduct a systematic review of open-source EHR systems based on the requirements and limitations of low-resource settings.

**Methods:**

First, we reviewed existing literature on the comparison of available open-source solutions. In close collaboration with the University of Gondar Hospital, Ethiopia, we identified common limitations in poor resource environments and also the main requirements that EHRs should support. Then, we extensively evaluated the current open-source EHR solutions, discussing their strengths and weaknesses, and their appropriateness to fulfill a predefined set of features relevant for low-resource settings.

**Results:**

The evaluation methodology allowed assessment of several key aspects of available solutions that are as follows: (1) integrated applications, (2) configurable reports, (3) custom reports, (4) custom forms, (5) interoperability, (6) coding systems, (7) authentication methods, (8) patient portal, (9) access control model, (10) cryptographic features, (11) flexible data model, (12) offline support, (13) native client, (14) Web client,(15) other clients, (16) code-based language, (17) development activity, (18) modularity, (19) user interface, (20) community support, and (21) customization. The quality of each feature is discussed for each of the evaluated solutions and a final comparison is presented.

**Conclusions:**

There is a clear demand for open-source, reliable, and flexible EHR systems in low-resource settings. In this study, we have evaluated and compared five open-source EHR systems following a multidimensional methodology that can provide informed recommendations to other implementers, developers, and health care professionals. We hope that the results of this comparison can guide decision making when needing to adopt, install, and maintain an open-source EHR solution in low-resource settings.

## Introduction

### Electronic Health Records

The world has been moving toward global health and equity in accessing health care services for many decades [1-3]. However, the majority of low- and middle-income countries (LMICs) still face challenges in providing comprehensive medical care compared with the developed countries. Many factors influence health care services in low-resource settings such as the lack of financing, absence of health care policy, and limited technical and human resources [4-9]. As a result, the adoption of eHealth in poorly resourced areas such as LMICs is a challenging task because of their limitations and regional specificities. To bring positive changes to public medical care in less developed economies, the World Health Organization recommends the adoption of electronic health (eHealth) solutions starting from national eHealth strategy [10] and electronic health records (EHRs) [11].

As information and communication technologies have rapidly become involved in health care, particularly in patient management, this has increased the efficiency and effectiveness of services provided by medical care facilities [12-14]. The EHRs era began in the middle of the 1960s when health care professionals changed the direction of patient management toward digital format. EHR systems not only increase accuracy and reduce mistakes through allergy alerts, access to laboratory data, and immunization history but also improve organizational and societal outcomes [13]. Moreover, the collected patients’ data can be used in research [15-19], giving an opportunity to study diseases and extract knowledge from clinical data. Despite the advantages of EHRs, the majority of developing countries cannot afford expensive proprietary software from big vendors and demand cost-effective solutions. Thus, the adoption of open-source EHR (OS EHR) is a possible solution for LMICs. Moreover, it is important to consider other accompanying expenses such as power supply, Internet connection, staff training, and system support.

One of the pioneers of OS EHR systems was the Veterans Information Systems and Technology Architecture (VistA) developed by the US Department of Veterans Affairs implemented in the late 1970s [20]. The system is still functioning and serves several million patients in US hospitals. Almost four decades after the first OS EHR system was introduced in developed countries, it has reached the developing world too [21].

### eHealth in Low-Resource Settings

In several studies conducted on eHealth adoption in LMICs, most were focused on EHR implementation [22-32]. Aminpour et al [33] pointed out that open-source EHRs have been widely used by resource-limited regions in all continents, particularly in Sub-Saharan Africa and South America. The authors concluded that HIS create opportunities to improve national health care, particularly in developing countries with minimal financial resources.

The major obstacles to OS EHR utilization in low-resource settings were discussed by Fraser and Blaya [5] after their experience in developing countries. The authors shared lessons they learned, highlighting key ideas such as the need for clinical staff involvement to provide qualitative data entry, the reuse of validated EHR implementations, and planning of offline data entry, which is particularly relevant because of an unstable power supply and Internet connection.

In studying the success criteria of OS EHRs for resource-limited settings, Fritz et al [24] concluded that a system’s functionality and technical infrastructure play an essential role, whereas financing is not a major aspect because of donors’ sponsorship in developing countries. The authors also mentioned the importance of organizational facets such as training availability, project management, and staff involvement.

Other studies focused on the regional distribution of OS EHR in developing countries, which included Africa [34-36], Latin America [37], and Asia [38,39]. Several studies focused on applying health record systems to concrete diseases such as human immunodeficiency virus (HIV) [40,41], tuberculosis [42], and diabetes [43]. For instance, Manders et al reported on their experience in the adaptation of an open-source health record system in a hospital in Mozambique.

The study by Tilahun et al [44] in Ethiopia was based on interviewing health care professionals in 5 hospitals. The findings showed clinical staff’s dissatisfaction, for example, with low service quality and entering data twice (both on paper and electronic medical records [EMR]). The authors suggest focusing on training, user support, and providing enough computers to increase the use of EMR systems by health care professionals.

### Open-Source Solutions

In a recent comparison of OS EHR systems [45-47], the authors used a multicriteria selection approach to compare and score the tools. The authors examined 13 chosen systems and compared 25 of their features.

One of the biggest open-source software (OSS) communities in health care is the Open Medical Record System (OpenMRS) led by Regenstrief Institute and Partners in Health; it focuses on the development of an electronic medical record solution to be used in resource-constrained environments. Initially, the system was implemented in Kenya and then was rapidly adopted by other health care organizations [35,48-58]. Starting from a simple data model, currently, OpenMRS has become an EHR platform with developers all over the world [59], contributing to its Java-based service-oriented architecture [60].

Bahmni is an integrated clinical software, which combines three open-source platforms: (1) OpenMRS for patient records, (2) OpenELIS (OpenELIS Foundation) for laboratory management, and (3) OpenERP (Odoo SA) for hospitals’ accounting operations. Bahmni was implemented by the ThoughtWorks company in 2012. The main goal of the software is to provide a universal solution for health care institutions. Bahmni has been deployed mainly in low-resource settings such as India, Bangladesh, and Nepal [61]. However, the number of implementations is growing and spreading to other regions [62].

GNU Health (GNU Solidario) is another OS EHR system that provided hospitals with a wide range of functionalities for patient management. The system was introduced in 2008 and has been adopted in several developing countries such as Paraguay, Kenya, the Philippines, and Bangladesh [63-66].

Open Source Clinical Application Resource (OSCAR) is a nonproprietary EHR system, developed by McMaster University, Canada. It is licensed under the GLv2, allowing other programmers to reuse the system code by acknowledging copyright and giving access to their modifications under the same license agreement. Unlike many EHR tools, OSCAR has been implemented mainly by doctors rather than developers. However, it has become widespread among health care providers [67].

OpenVistA (Medsphere Systems Corporation) is another OS EHR system based on the VistA software [55], one of the first EHR systems implemented for American veterans. OpenVistA allows multiple clinicians to simultaneously access various patient data in real time. The system provides progress notes, various templates, ordering and reporting tools, audit capabilities, electronic signature, document management, and data integration tools, among other features of OpenVistA.

FreeMED (FreeMED Software Foundation) is a modular open-source system for medical data and was implemented almost two decades ago in the United States. It also has an external billing subsystem called REMITT.

OpenClinic GA (Medical eXchange Solutions) is an open-source system that includes EHR, laboratory management, and pharmacy management. It was implemented in 2006 in Belgium and has been used in many countries worldwide. The system has been used in several resource-limited settings, including hospitals in Rwanda, Mali, and Burundi.

Overall, there are many open-source projects available for health care providers. However, we selected five OS EHR systems to make detailed research of each and focus on the functionalities provided, which are pointed out in the next section.

## Methods

### Identification of Key Features

In this section, we reviewed OS EHR systems discussing their strengths and weaknesses according to a set of predefined parameters. The evaluation methodology was defined in close collaboration with the University of Gondar Hospital, Ethiopia, and it included two main aspects: (1) user requirements and (2) systems’ selection.

The selection of the most important features of these systems was performed through interactive discussion sessions with experienced physicians from Gondar and partners from the EUROLEISH-NET research network, with great experience in low-resource settings, both in Asia and in Africa. In this phase, the health care professionals’ help was crucial because of the specificity of medical workflows and specialized vocabularies. Moreover, we analyzed previous publications related to eHealth development in LMICs, where the authors also tackled the specificity of the infrastructure and services. From this study, we obtained the following set of features:

1. Integrated applications referring to the type of subsystems that are integrated in each solution
2. Configurable reports, general report templates may be generated by an end user, without programming skills
3. Custom reports, specific report templates can be created by a user with basic programming skills
4. Custom forms allow making a form template to gather patient data or other clinical information
5. Interoperability, ability to exchange data with other applications (it includes the support for standards such as Health Level-7 [HL7], Fast Healthcare Interoperability Resources [FHIR], and Digital Imaging and Communications in Medicine [DICOM])
6. Coding systems, referring to the utilization of medical terminology classification, such as SNOMED (Systematized Nomenclature of Medicine) or International Classification of Diseases (ICD)-10
7. Authentication methods enable users to access systems without credentials by using the authentication from third-party application (eg, single sign-on, lightweight directory access protocol [LDAP])
8. Patient portal can be accessed by patients to consult their own records
9. Access control model, management of users’ access, roles, and permissions
10. Cryptographic features refer to applying security techniques to conceal patient data
11. Flexible data model can cope with various and dynamic data characteristics
12. Offline support, still operating on the client side, when disconnected from the server
13. Web client, the application can be accessed through a common Web browser
14. Native client, has a client that runs natively on a specific desktop platform (eg, in Windows or Linux)
15. Other clients refer to more than one client version to cope with hardware limitations
16. Code-based language, programming language used for system’s source code (eg, Java)
17. Development activity refers to how often the software is updated and to the developers’ support
18. Software modularity refers to software engineering quality, namely, how manageable the system is
19. User interface refers to the usability of the system and how the user interface is perceived by end users
20. Community support includes discussion forum activities and community contributions such as translation of user interface to other languages
21. Customization, how easy modifications are without rewriting the core code of a system to reach established requirements

We ranked several features such as modularity, user interface, community support, and custom forms to show our evaluation of the EHR systems. The modularity rank, for instance, highlights whether the platform architecture is well thought out to simplify future modifications and additions from developers. Community support is another important aspect of open-source software that helps volunteer developers and IT teams to improve and share their contributions.

### Software Selection Process

**Figure 1:**
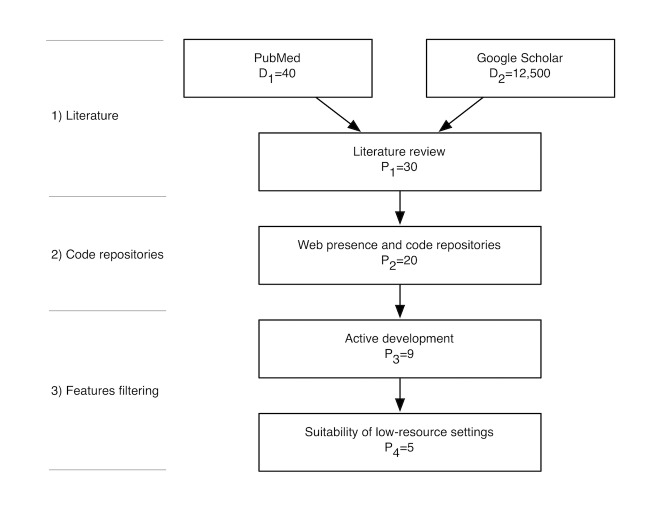
Flowchart of the open source electronic health record selection process.

The selection of open-source EHR software for this review was based on a representative set of open-source EHR systems found in the literature, as well as popular and active projects in open-source repositories. The methodology, which is depicted in Figure 1, included the following steps: (1) literature, (2) code repositories, and (3) features filtering.

In the first step, we searched on PubMed for papers referring to OS EHR software using the terms “open source” and “EMR or EHR,” combined with the MeSH term “computer software.” We validated the content of the retrieved publications leading to a total of 40 documents (D_1_=40). We also queried Google Scholar using an advanced search composed by the term “software” combined with the exact expression “open source” and containing at least one of the terms “electronic medical record, electronic health record, EHR, and EMR.” From the returned results (D_2_=12,500), we evaluated the top 100 documents to verify its relevance to our study. From this assessment, we identified 30 potential solutions (P_1_=30).

In the second phase, a common Web search engine (Google) was used, mainly for obtaining information about EHR solutions, for example, project status and resources. For each, we gathered the availability of public websites (24) and public source code repositories (23), from which we ruled out the ones outside the intersection of these (P_2_=20).

The last was the filtering step. First, we excluded the projects with lower activity (code updates, discussion forums, case studies, etc), which resulted in a more reduced set (P_3_=9). For each of these solutions, we assessed their capability to fulfil the limitations of a low-resource setting (eg, localization support and cross-platform support), ending up with the following group (P_4_=5): GNU Health, OpenEMR, FreeMED, OpenMRS, and Bahmni.

The selected systems were then installed and extensively evaluated against the requirements previously discussed. In the following sections, the characteristics of these five systems are discussed and compared.

## Results

### GNU Health

GNU Health is an EHR software and health information system (HIS) developed by GNU Solidario, a nonprofit and nongovernmental organization for health and education development. The main areas of GNU Health supports are (1) individual management, (2) patient management, (3) health center management, and (4) information management.

Individual management refers to a person’s basic information, genealogy, household, domiciliary infrastructure, and so on. The GNU Health project emphasizes on the importance of social and economic development, with a strong focus on relevant factors such as level of education, occupation, living conditions, and family relations.

Patient management comprises the collection of different types of data related to a patient’s health, for instance, lifestyle parameters such as diet and exercise, addictions, sexuality, or safety. It also includes management of the patient’s health care regarding encounters and evaluations, medical procedures, laboratory test requests or results, and so on.

Health center management mainly comprises enterprise resource planning (ERP), for instance, financial and human resource management, sales, invoicing and accounting, inventory, stock, and supply chain management. It also features a focus on pharmacy and laboratory management. The managed subject can either be the institution as a whole or individual department.

Information management covers the tasks that combine data produced on the platform. This includes building reports or presenting demographics with configurable parameters, which are useful for epidemiology studies or crossing different data, for instance.

The areas explained above generally involve teams from different fields of activity in which members generally only work within their corresponding domain of information.

As the information stored by GNU Health is highly sensitive, the platform is developed with a focus on data confidentiality and integrity. It provides serialization, hashing, signing, and verification, which are commonly used in features that handle or produce sensitive information such as reports, prescriptions, or laboratory tests. It allows doctors to digitally sign death certificates or users to verify the authenticity of reports compiled in other GNU Health systems. This is achieved via a bundled client-side plug-in for pretty good privacy that uses GNU Privacy Guard implementation.

Most of GNU Health’s functionality is provided by the official add-on modules, which extend the core module. These are separated into general domains or specialties, for example, pediatrics, surgery, or genecology. Modules add new functionality to these domains by reusing models of given entities and creating specification classes designed in other modules. As this creates dependencies between modules, in order for a target module to work, its parent modules need to be installed as well. In GNU Health, the way to provide new functionality such as custom forms for collecting patient information is through developing new modules. As this requires programming knowledge, building and adding custom forms becomes a difficult task for nondevelopers.

**Figure 2:**
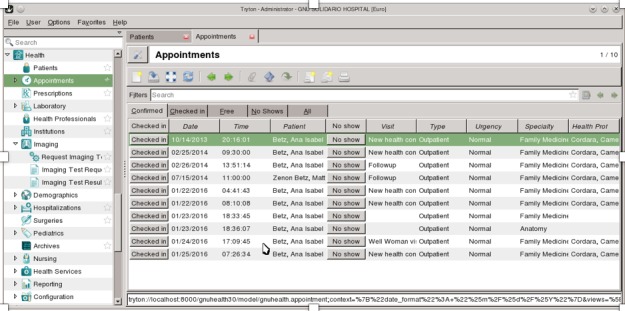
User interface of GNU Health’s client application.

GNU Health user interface contains a global menu where functionalities are presented in a tree-style structure (Figure 2). The menu supports filtering by text, providing easier and quicker ways to find the desired functionality. Several items can be active at the same time, making use of tabs for arranging the multiple content panels. It is common for some features to present a multitude of functionalities separated into their own tabs, adding to navigation and display complexity.

GNU Health client does not provide offline support, that is, interactions made in the client while the server is disconnected are lost. There is a workaround by deploying a GNU Health server on each client machine, synchronized in a centralized mode with the main server. Replicating sensitive data in each client device would raise complexity, security, and privacy issues and would also require different and more expensive hardware than that needed for running just a GNU Health thin client.

### OpenEMR

OpenEMR is an EHR software, medical practice management software (PMS), and ERP software mainly supported by OEMR 4, a nonprofit organization (NPO) whose goal is to ensure that all people have access to high-quality medical care through its software and services [68]. OpenEMR is licensed under GNU general public license (GPL) and comes with a great amount of functionality to cover a wide range of areas of an HIS in addition to patient records and management, including support for billing and claims management, ERP, and information management. The system is written in PHP, a scripting language that is widely supported and has cross-platform runtimes. The use of Web technologies makes the platform accessible through many different devices, provided they have a compatible Web browser.

With respect to patient records, clinical observations can be made through a variety of forms already provided. Observations captured on a patient’s different visits can be presented later through graphical charts or tables showing changes in values over time. Free-text type notes can be attached to a patient’s encounter and medical issues registered in a flexible system of coding, which supports Current Procedural Terminology (CPT), Healthcare Common Procedure Coding System (HCPCS), ICD-9, ICD-10, and SNOMED Clinical Terms (SNOMED CT) codes. In the prescription management area, doctors can register prescriptions and their frequency, which can then be printed, faxed, or sent electronically to a few supported third-party platforms. This can be complemented with the pharmacy dispensary module, which enables in-house drug dispensing with support for inventory, integration with drug databases, and stock tracking. In patients’ history, medical procedures, immunizations, and laboratory tests made can be registered along with results.

OpenEMR provides a robust calendar subsystem, which is capable of handling different types of events, such as appointments, notifications, alerts, and automated report generation. With this subsystem, it is possible, for example, to automatically send an email reminder to a patient each time he needs to take his medication, or automatically run reports of weight assessment for children and adolescents each month.

OpenEMR provides a native portal aimed at use by patients and other registered users, supporting integration with other external patient portals. The reporting subsystem comes with an extensible profile of reports that cover many of the platform’s different areas. Some of these are configurable, for instance, patient reports can include medical history, encounters, billing, communications, or other patient-related information. Adding custom reports to the OpenEMR platform is possible through the same method as the one used by the preloaded reports. These are built with PHP programming for which a specific PHP template for coding reports is provided. There is no alternative method to create custom reports; consequently, programming knowledge and other advanced computer skills are required for the creation of new reports.

For interoperability, OpenEMR supports the HL-7 standard, which is used for exchanging patient data between other systems, and the ANSI X12 Electronic Data Interchange standard, mainly used in billing and invoicing in the ERP.

The OpenEMR platform can support more than 20 languages and is compatible with Unicode Transformation Format (UTF)-8. The display language of the user interface is user dependent, meaning that multiple users can use the platform simultaneously in their desired languages.

**Figure 3:**
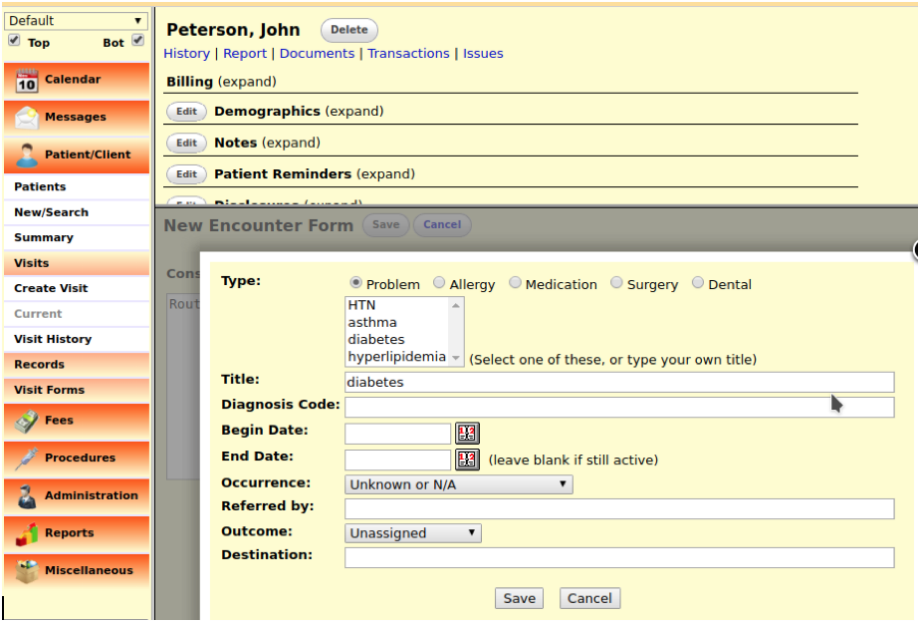
User interface of the OpenEMR platform.

User interaction with OpenEMR is via a Web interface (Figure 3). The user interface arranges active content in a dual panel mode. The first panel is always shown and is used to display primary content of the active area or context. The second panel either shows a given global functionality of the active context such as past encounters and documents while browsing the patient’s context or is used to show selected content in addition to the first panel. This proves useful for showing related information side by side but quickly builds up complexity in the interface. Some functionality also opts to open a separate frame inside the content panel, or even a new pop-up window to display or gather information, again adding complexity.

The OpenEMR project development is active, with an average of two major releases per year. The project has a strong community of users and backing companies, with many OpenEMR modules developed by third parties and contributed to OpenEMR, and these are now part of the project. The documentation has extensive user and implementation guides but falls short for developers.

### FreeMED

FreeMED is an EHR software and medical PMS by FreeMED Software Foundation 7 and is licensed under GPL. FreeMED has a modular architecture that is designed with functionality and interface separation, which allows customization or development of modules without having to rewrite core components of the system.

A patient record module contains personal and demographic information. Preloaded templates are provided, which can be used for collecting clinical observations during patient visits. Medical assistance is provided by the diagnosis family module and can be coded using CPT and ICD codes. It is possible to assign medication to a patient and manage a registry with them. Patients can be arranged in groups that are assigned to doctors or referenced in programs. There is an area for triage and call-ins useful for registration secretaries, which can be complemented by the appointment scheduling subsystem. Appointments can be scheduled for either an individual or groups of patients, and the interface has safeguards for double booking, presenting warnings in case 2 patients get booked for the same provider at the same time.

FreeMED uses HL-7 for the exchange of medical data. This support is obtained via integration with Mirth Connect, a cross-platform HL-7 interface engine.

FreeMED already comes with some configured reports, but adding new ones requires related technical skills and also knowledge of the FreeMED data model. The reporting subsystem is dependent on the Java library Jasper Reports for report generation. There is a lack of documentation on how to add custom forms to FreeMED. User interaction with the platform is through a Web interface. After language selection and user authentication, different functionalities are displayed depending on the user’s access clearance. Access is controlled with Access Control List (ACL), in the same way as described previously for OpenEMR, also making use of the phpGACL library.

**Figure 4:**
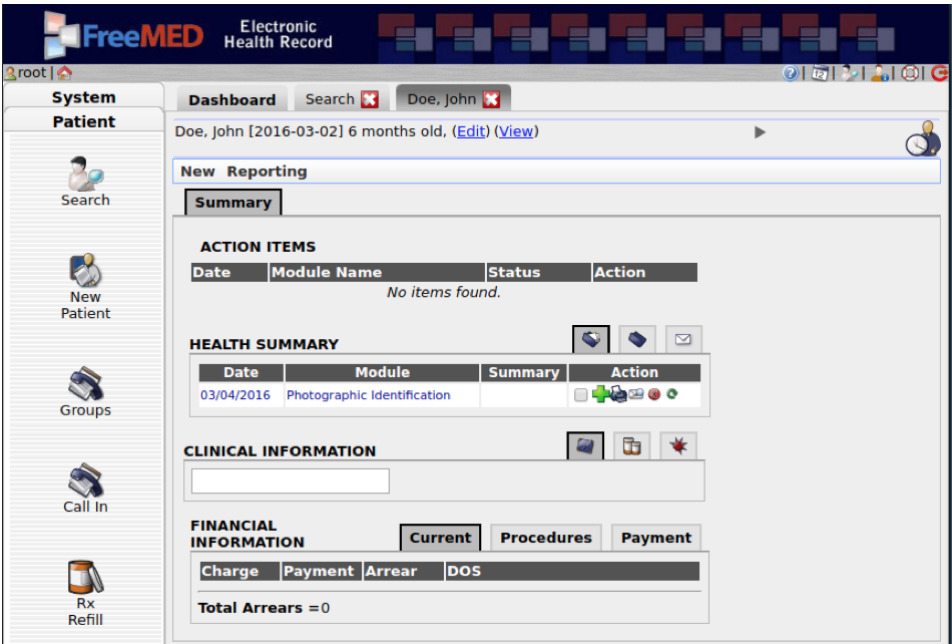
User interface of the FreeMED platform.

The user interface has an always-present menu, which groups functionalities in categories (Figure 4). The active functionality is displayed on a content panel. Several items can be opened at the same time, being grouped into tabs that keep its current state in the background without interrupting the user experience. The content has a clean presentation, and while some more complex features may show different functionalities grouped into their own tabs, there is an option to choose the arrangement between tabbed and flat presentation.

FreeMED is written mostly in PHP and Perl. Additionally, it requires a Java runtime environment for the report subsystem and JavaEE for REMITT, as they are written in Java. As all the technologies used in the software are cross-platform, FreeMED can be run on different operating systems, ranging from POSIX-compatible such as Linux or BSD to Microsoft Windows. The software is designed in a client-server application model. The platform does not provide offline support, so if the communication is interrupted between the client and the server, users need to wait for the reestablishment of the connection. Akin to OpenEMR, access to the database is through the ADOdb PHP library, which allows transparent usage of many different database management systems (DBMS). Nevertheless, parts of the code base are not DBMS-agnostic, and thus, the project recommends using MySQL.

FreeMED supports localization, and the project claims multiple languages support in addition to English, including German, French, and Polish. There is also an online public project to contribute translations, which has already attained complete localization in Portuguese and Russian [69]. FreeMED supports multiple ISO character set formats and stores entry data with it, making it possible to maintain a demographic database in one language and ISO set, independent of the localization that displays.

The source code is available on a public repository website, which also provides a brief installation guide and a Virtual Machine image of FreeMED for demo purposes. There are no development guides, and the project lacks proper documentation overall. There is also a FreeMED demo available online, but there are no user guides on the official site or in the FreeMED software itself.

### OpenMRS

OpenMRS is an EHR software led by two collaborative NPOs, Regenstrief Institute and Partners in Health. One of the main objectives of the project is to build a robust solution for health care with a special focus on resource-constrained environments. OpenMRS is a highly modular platform, meaning that features are external to the platform and added through add-on modules. An application programming interface (API) plays a major role in this by allowing access to OpenMRS platform functionality. The platform core uses a centralized dictionary of concepts, a customizable part of OpenMRS that strongly defines each implementation. In the dictionary, concepts are metadata of given entities, defined by name, data type, appropriate attributes, and relationships to other concepts. These are used as models to be instanced later in some user form or report. For example, a value collected during a patient encounter is an instance of a given concept that takes the form of an observation. As a model, concepts work as the building blocks that describe forms, orders, clinical summaries, reports, and so on.

**Figure 5:**
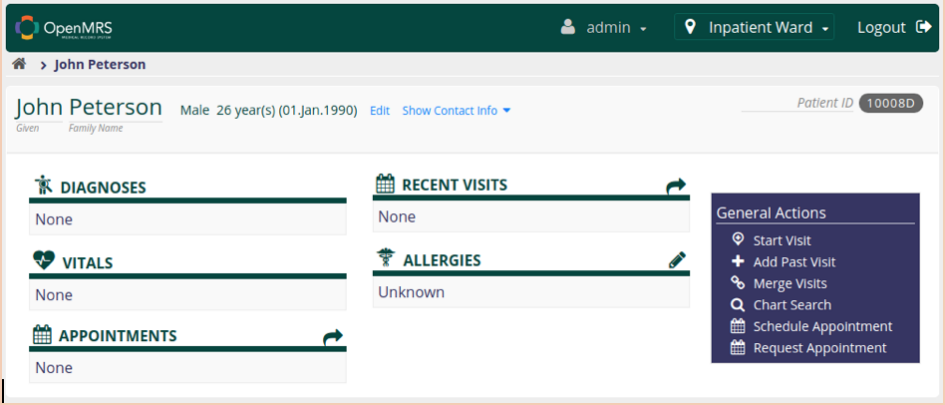
OpenMRS’ modern user interface.

OpenMRS is officially released in two different distributions, one which contains just the OpenMRS platform, and another called the reference application, which packs selected modules that extend and add features to the platform. The framework standardizes modules and defines their interface presentation, communication, behavior, and security, enabling the creation of an OpenMRS application ecosystem. Through modules, OpenMRS extends its features, providing appointment scheduling and management, an event system for notifications, a subsystem for allergy registration, representation of patient observations through charts, and so on (Figure 5).

OpenMRS has a robust subsystem for the creation of custom forms. Any form relies on a schema that defines the fields and concepts it will use, which are supported by the concept dictionary. Although custom forms can be created and their form schema can be designed from within OpenMRS legacy user interface, the methods for designing the forms are independent of the platform.

A reporting subsystem allows three ways of building reports, that is, indicator reports, row-per-patient reports, or custom reports. A report is built through associations with dataset definitions that act as a key to values to include in the report. The dataset can generally refer to person or patient properties and similar attributes that contain only one recorded value stored since its last entry, commonly used in row-per-person reports. The combination of values from multiple observations is possible and is used for building indicator reports. A report definition can be programmed to filter (cohort) a defined set of patients for use as the input for report generation.

Access management follows a role-based access control (RBAC), which covers both the user interface and access to OpenMRS API via REST services. Importing and exporting metadata between platforms can be performed either manually or automatically, using the metadata sharing module. The module is advanced enough to provide automatic synchronization mechanisms, with support for record merging and conflict resolution.

OpenMRS has native support for the HL-7 standard and can export data directly to other applications. There is also support for DICOM and radiology standards via a third-party module, in conjunction with dcm4chee, a medical imaging archive and manager. Native support for FHIR is also present natively in the platform from version 2.0.

Officially, the project provides an extensive *wiki* with features content for developers, implementers, and end users. Two online demos are provided, one of the reference application and another with a demo for management of drug-resistant tuberculosis, which can show the level of customization that OpenMRS offers. Currently, more than 175 separate modules are available, but their compatibility depends on the core version because of changes in the OpenMRS API.

OpenMRS has full UTF-8 support and some extra localizations in addition to English, with the possibility to add more. The project provides an online portal for collaborative translation of its user interface, reference application, and commonly used third-party modules [57].

### Bahmni

Bahmni is a hospital system for low-resource settings developed by ThoughtWorks Global Health under an AGPL license. Bahmni is built through separate OpenMRS modules, and its user interface communicates with these using the OpenMRS API via REST. The system incorporates most of the OpenMRS platform functionality and extends it with support for picture archiving and communication system (PACS), laboratory information system (LIS), and ERP features. This is done via integration with three separate applications: dcm4chee, which provides the first; OpenELIS, which provides the second; and OpenERP (currently named Odoo), which provides the third.

In a registration process, information such as identity, demographics, photograph, contacts, and other socioeconomic details of a patient can be captured and associated with a person via a newly generated patient identifier. Different types of relationships can be registered, including genealogical or patient-provider relationships. When a patient registers with an institution, there is a strong possibility that he already has health-related documents stored in other institutions, for example, record scans, x-ray images, or other types. Bahmni EHR supports storing these within the system and attaching them to a patient’s profile. A patient’s profile is displayed through a modular dashboard, where customizable widgets display the most recent information from several different categories of the patient’s medical records. These can be, for instance, laboratory tests and results, programs enrolled in, or last visit observations. From observations made in several encounters, the history of changes can be presented.

**Figure 6:**
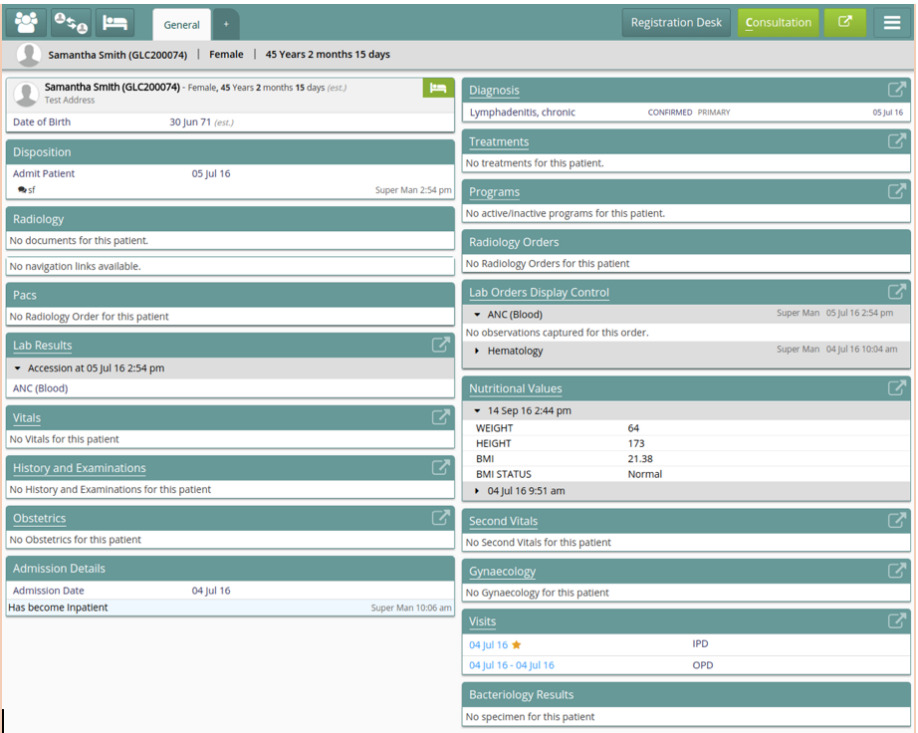
Bahmni patient dashboard.

Clinical services are centered around the patient’s profile. These services become available in the consultation area of the dashboard after a patient is active in the system, that is, the patient is in a given encounter with a provider. From here, several types of services are provided: capturing observations, making laboratory or radiology orders, registering diagnosis and medication, managing patient disposition, or taking consultation notes (Figure 6).

With respect to clinical observations, Bahmni already provides multiple forms by default. The form subsystem is very customizable and can easily be extended with more forms. These can be built by creating and changing specific concepts of the dictionary. The form’s layout and other parameters can be defined in JSON and, if necessary, there is support for integration with a server-side framework for advanced customization of a form’s logic and other aspects. Current and past diagnosis can be associated with a patient and registered along with attributes such as order, confidence, and status. Diagnosis made can also be mapped to ICD-10 codes.

Regarding laboratory orders, Bahmni provides a panel of tests divided in several categories. The laboratory subsystem is flexible, making it possible to choose just the needed tests and categories or arrange them all together. New laboratory tests can be easily created and added just by using the concepts dictionary.

Radiology orders containing patient and investigation details can be requested within Bahmni EHR, which are sent to a modality via HL-7. The results are integrated with the patient profile even if they come as native unsupported formats, in which references to them are shown. For instance, results that come as DICOM images are archived in an accessible PACS and referenced from the patient profile from which they can be opened in compatible DICOM viewers.

Medication management includes registration of past and current medication from a customizable drug registry, which has the route, frequency, dosage, and instructions for each. There is a drug dispensing service, which is integrated with OpenERP and supports in-house dispensing with segregation of stocks. Patient disposition refers to a patient’s state usually set at the end of a provider encounter.

OpenERP is used predominantly for sales and purchase management, including inventory and accounting, and it is integrated to some extent in Bahmni EHR. For instance, registration fees or payment of drugs dispensed is registered in OpenERP under patient billing and inventory.

The reporting area comes with many preloaded reports, which can be run within a given period and exported to CSV, PDF, HTML, or Excel formats. Additionally, the platform provides integration with Jasper Reports, giving support for custom, flexible, and more varied reports.

The access control on Bahmni EHR is provided by the OpenMRS platform. However, there are new access roles associated with the new functionalities and services of Bahmni EHR. Additionally, user access can now be set to use two-factor authentication.

Bahmni EHR supports offline network alerts by displaying a notification in the user interface when the connection is down. This is helpful to avoid loss of filled-in data by submitting it unknowingly when a connection to the server is down. True offline support is also provided via a browser extension or a mobile app, offering the possibility of filling in forms and other user interface functionalities without an active connection to the server. The data produced with this functionality are saved locally on the device and can be uploaded later. The community is closely tied with the OpenMRS community as both come under the same forum and are very active.

**Table 1:** Matrix with several aspects of the evaluated electronic health records software. Some features were evaluated according to a ranking that varies between 1 (low) and 3 (high).

Feature or system		GNU Health	OpenEMR	FreeMED	OpenMRS	Bahmni
1	Integrated applications	EHR^a^, HIS^b^	EHR, PMS^c^, ERP^d^	EHR, PMS	EHR	EHR, PMS, ERP, LIS^e^, PACS^f^
2	Configurable reports	Yes	Yes	No	Yes	Yes
3	Custom reports	No	No	No	Yes	Yes
4	Custom forms	-	1	-	3	3
5	Interoperability	FHIR^g^, custom	HL7^h^	HL7, DICOM^i^	HL7, DICOM, FHIR	HL7, DICOM, FHIR
6	Coding systems	ICD-10^j^	ICD-9/10, SNOMED^k^, CPT^l^, HCPCS^m^	ICD-10, CPT, LOINC^n^, ATC^o^	CIEL/MVP^p^, LOINC ICD-10	CIEL/MVP, ICD-10, SNOMED
7	Authentication methods	LDAP^q^	LDAP, AD^r^	-	-	-
8	Patient portal	No	Yes	No	No	No
9	Access control model	RBAC^s^	ACL^t^	ACL	RBAC	RBAC
10	Cryptographic features	Sign, encrypt	Encrypt	-	-	-
11	Flexible data model	No	No	No	Yes	Yes
12	Offline support	Yes	No	No	No	Yes
13	Web client	Yes	Yes	Yes	Yes	Yes
14	Native client	Yes	No	No	No	No
15	Other clients	Yes	No	No	No	Yes
16	Code-based language	Python	PHP	PHP	Java	Java
17	Development activity	3	3	2	3	3
18	Software modularity	3	1	2	3	3
19	User interface	2	1	3	2	3
20	Community support	3	3	1	3	3
21	Customization	1	2	1	3	3

^a^EHR: electronic health record.

^b^HIS: health information system.

^c^PMS: practice management software.

^d^ERP: enterprise resource planning.

^e^LIS: laboratory information system.

^f^PACS: picture archiving and communication system.

^g^FHIR: Fast Healthcare Interoperability Resources.

^h^HL7: Health Level-7.

^i^DICOM: Digital Imaging and Communications in Medicine.

^j^ICD: International Classification of Diseases.

^k^SNOMED: Systematized Nomenclature of Medicine.

^l^CPT: Current Procedural Terminology.

^m^HCPCS: Healthcare Common Procedure Coding System.

^n^LOINC: Logical Observation Identifiers Names and Codes.

^o^ATC: Anatomical Therapeutic Chemical.

^p^CIEL/MVP: Columbia International eHealth Laboratory/Millennium Villages Project concept dictionary.

^q^LDAP: lightweight directory access protocol.

^r^AD: Active Directory.

^s^RBAC: role-based access control.

^t^ACL: Access Control List.

## Discussion

### Systems' Comparison

The above assessment allowed us to undertake a systematic review of five popular EHRs that can be used in low-resource settings without being an economic burden. Due to constraints in LMIC and according to the workflow of health care institutions, it is important to consider key operation functionalities and the adaptability of each system. Table 1 summarizes these key aspects, presenting for each system the characteristics defined in our evaluation methodology.

### Conclusions

Despite the wide adoption of EHR and eHealth solutions in many developed countries, the scenario is quite unbalanced when compared with health digitalization in low-resource settings. This work was performed in close collaboration with the Hospital of Gondar, Ethiopia, with which the evaluation methodology was defined. We conducted a local assessment of the used solutions and identified a scenario that is very dependent on IT solutions from nongovernmental organizations, which, despite their good will, led to partial and incompatible solutions. There is a clear demand for open-source, reliable, and flexible EHR systems in these health care facilities. Hence, the process of selecting a suitable solution that covers their needs is a central task. In this study, we have evaluated and compared five open-source EHR systems following a multidimensional methodology. We hope that the results of this comparison can guide decision making when needing to adopt, install, and maintain an open-source EHR solution in low-resource settings.

## Abbreviations

ACL: Access Control List
ATC: Anatomical Therapeutic Chemical
API: application programming interface
CIEL/MVP: Columbia International eHealth Laboratory/Millennium Villages Project
CPT: Current Procedural Terminology
DBMS: database management systems
DICOM: Digital Imaging and Communications in Medicine
eHealth: electronic health
EHR: electronic health record
EMR: electronic medical record
ERP: enterprise resource planning
FHIR: Fast Healthcare Interoperability Resources
GPL: general public license
HCPCS: Healthcare Common Procedure Coding System
HIS: health information system
HIV: human immunodeficiency virus
HL7: Health Level-7
ICD: International Classification of Diseases
LIS: laboratory Information system
LDAP: lightweight directory access protocol
LMICs: low- and middle-income countries
LOINC: Logical Observation Identifiers Names and Codes
NPO: nonprofit organization
OpenMRS: open medical record system
OS EHR: open-source EHR
OSS: open-source software
OSCAR: open source clinical application resource
PMS: practice management software
RBAC: role-based access control
SNOMED-CT: Systematized Nomenclature of Medicine-Clinical Terms
UTF: Unicode Transformation Format
VistA: Veterans and Information System &Technology Architecture
